# Protocol for a feasibility study of a self-help resource based on the principles of cognitive behavioural therapy to reduce needle fear in young people aged 11- to 16 years

**DOI:** 10.1186/s40814-026-01893-9

**Published:** 2026-08-14

**Authors:** Fiona Sotir, David White, Gemma Wheeler, Helen Rodd, Zoe Marshman

**Affiliations:** 1 School of Clinical Dentistry, University of Sheffield, Sheffield, UK; 2 Clinical Trials Research Unit, Sheffield Centre for Health and Related Research, Sheffield, UK; 3 NIHR HealthTech Research Centre in Paediatrics and Child Health, Sheffield, UK

**Keywords:** Child needle fear, Feasibility study, Self-help resource, Cognitive Behavioural Therapy, Protocol

## Abstract

**Background:**

Needle fear affects up to half of all young people, with the potential to impede their accessing and acceptance of healthcare interventions. There is presently a lack of accessible self-help resources available to support young people in managing needle-based procedures. This feasibility study aims to create a trial protocol for future definitive assessment of a self-help tool co-designed with young people, to manage their fear of needles.

**Methods:**

This article presents the protocol for a study to evaluate the feasibility of a future definitive randomised trial, and broad acceptability of a developed self-help resource. In total sixty children aged 11–16 years will be recruited from across three sites (secondary dental care, secondary medical care and a school setting) and directed to complete the proposed outcome measures and access a self-help resource developed by the authors in collaboration with young people, parents and healthcare professionals in order to test feasibility. Additionally, a subset of 20 children will be invited to participate in qualitative interviews following their involvement in the feasibility phase to explore their experiences, any key facilitators or barriers, and their general acceptance of the resource. This study will address key uncertainties around recruitment of sites and participants, and variability of outcome measures, prior to a definitive future trial.

**Discussion:**

The resource was developed in collaboration with young people, parents and healthcare professionals. This self-help resource has the long-term potential to benefit young people through support in managing their fear of needles. This study will provide methodological evidence to support a future definitive trial evaluating the effectiveness of the resource.

**Supplementary Information:**

The online version contains supplementary material available at https://doi.org/10.1186/s40814-026-01893-9.

## Background

Needle fear, a phrase used in this protocol to describe a continuum from mild fear to true phobia towards the medical use of a needle, is common in young people, affecting up to 50% according to a recent estimate [1]. It can affect young people in many ways, interfering with their ability to accept many types of needle-based procedures. Difficulty in accepting venepuncture, cannulation, intra-oral injections and vaccinations could cause negative impacts to the health of a young person through delays in diagnosis, difficulty accepting care, worsening of disease and reliance on pharmacological methods such as sedation or general anaesthetic, which often carry higher risks and have longer waiting times [2–6]. Children being cared for in hospital settings have reported that procedures involving needles are the most fear-inducing of all procedures they experience [7].

Young people with fear of needles are also a known source of occupational stress to the healthcare professionals who treat them [8–10]. It is also probable that the management of these patients has impacts on the wider health service. They may account for higher numbers of missed or cancelled appointments, often require additional appointments to treat, are more likely to be referred for pharmacological management and to be referred into secondary care settings. These impacts would all cost the National Health Service (NHS) additional time and resources compared to young people with no fear of needles.

Secondarily to impacts on health and well-being, the fear of needles can also be a barrier to children who wish to participate in clinical research. It is recognised by the Royal College of Paediatric and Child Health Ethics Advisory Committee that some children may find the process too distressing to allow participation [11]. Parents may also be reluctant to consent to blood samples being taken from their children for research purposes, considering that this will cause unnecessary pain and distress [12].

Management of anxiety, particularly self-help options, has become more prevalent over the course of the last decade. A Cochrane systematic review in 2015 found that cognitive behavioural therapy (CBT) could be highly effective for managing anxiety and related disorders in children [13]. CBT can be defined as a therapy based on the theory that thoughts, feelings, behaviours and symptoms are all connected and play a role in maintaining an individual’s fear. The process supports individuals to identify the unwanted thoughts and behaviours which could influence their fear and identify strategies to mitigate these. [14]. This technique has proven success at reducing needle fear (injections) when provided by a trained health professional, such as a psychologist [15, 16]. However, in the UK access to CBT in NHS Child and Adolescent Mental Health Services can be difficult due to long waiting lists and strained services. As part of the stepped care model, patients with lower levels of anxiety are often offered alternatives prior to referral to secondary care psychological services. These alternatives may obviate the need for onward referral or may provide a starting point for patients prior to more intensive treatment. The National Institute for Health and Care Excellence (NICE) guidance on Common Mental Health Problems (CG123, [17]) recommends that individual self-help CBT models should be considered for these patients. Additionally, studies focusing on the costs of CBT when provided for people with anxiety disorders such as depression or obsessive–compulsive disorder have shown that it is cost-effective over the individual’s lifetime [18], and therefore could be even more beneficial if such treatment were provided at an earlier stage. While a self-help CBT intervention has been found to reduce dental anxiety in children [19, 20], there appears to be no equivalent self-help resource for the management of needle fear. Although at present no specific evidence exists demonstrating the effectiveness of self-help CBT for reducing needle fear in children, the current evidence would support the hypothesis that the approach could be successful.

### Aims

This study will investigate the feasibility of evaluating a self-help resource based on the principles of CBT in reducing the fear of needles in young people (aged 11–16 years) in a future definitive randomised controlled trial (RCT).

It will address the following methodological uncertainties:

1. Feasibility of recruiting participants across sites
2. Feasibility of implementing study protocol at proposed sites
3. Variability of candidate primary outcome measures for a future definitive trial
4. Willingness of participants to be randomised, and of clinicians to recruit
5. Acceptability of resource

## Methodology

An uncontrolled mixed-methods study will be conducted across three sites in two stages in order to meet the aims and capture the perspectives of young people and healthcare professionals. Ethical approval has been granted by an NHS Proportionate Review process (22/PR/1451).

## Resource

The self-help resource, based on the principles of the five areas model of CBT [21], was developed through a co-design collaboration with young people, families and healthcare professionals. It is an online website containing information and advice about anxiety, needle-based procedures, and coping strategies for both young people and their parents. The most recent version of the resource can be accessed at: https://www.sheffield.ac.uk/dentalschool/our-people/academic-staff/fiona-sotir. It also contains a communication tool for use with healthcare professionals. Young people will be directed to the resource during the study, and will be able to use it unsupervised, although they may choose to access it with their parent/carer. The resource can be accessed as the young person wishes; there is no minimum or maximum time limit imposed.

## Quantitative component

### Settings and sample size

The study will be run in three sites:

1. Healthcare–secondary care medical
2. Children’s hospital setting
3. Multiple clinics including those having treatment under general anaesthetic
4. Procedures including cannulation, venepuncture, subcutaneous or intramuscular injection
5. Healthcare–secondary care dental
6. Dental hospital setting
7. Paediatric dentistry clinics including those having treatment with local anaesthetic, sedation and general anaesthetic
8. Procedures including intra-oral injection, cannulation or venepuncture
9. Schools
10. Secondary school setting
11. Children undergoing human papillomavirus vaccination

The sample size for the quantitative element of the study is 60 participants in total, with the intention of recruiting 20 participants from each site. The proposed primary outcome for a future definitive trial is young people’s self-reported needle fear. Participants will be directed to complete questionnaires containing three candidate primary outcome measures, at two timepoints (before and immediately prior to treatment) and to use the resource. The candidate outcome measures are related to needle fear: a Visual Analogue Scale (VAS), the Children’s Fear Scale (CFS, [22]) and the Injection Phobia Scale-Anxiety (IPS-Anx, [15]). The sample size will provide methodological evidence for the feasibility of recruiting participants from each of the settings and the variability of the candidate primary outcome measures. This sample size will be sufficient to assess this with a reasonable degree of precision and is in line with the median target sample size for UK feasibility studies [23] and the generally recommended sample sizes [22, 24]. It will also facilitate the recruitment of an adequately sized, heterogeneous and purposive sub sample for the qualitative element of the study.

### Recruitment

Suitable participants will be identified through a screening letter where they will answer a single question: ‘How do you feel when you need to have treatment with a needle?’. The answer options provided are: ‘Very worried’; ‘A little worried’; ‘Not at all worried’ or ‘Prefer not to say’. Those who select either ‘Very worried’ or ‘A little worried’ will be suitable for inclusion. The screening letter will be provided in person to young people attending assessment appointments in healthcare settings. Within the school settings, the young people attend an assembly where they will be provided the letter as part of a recruitment pack containing all initial study documents. Letters will be returned directly to the researcher either in person (healthcare settings) or by returning them through the school office.

Potential participants will be provided with participant information sheets, which have been designed with children and young people alongside a graphic designer, with a separate information sheet for parents and carers.

### Procedures

In healthcare settings, participants who choose to take part will complete assent forms and their parent or carer will complete a consent form. The participants will then be provided with a baseline questionnaire which they will be asked to complete. They will be provided with a second questionnaire to complete immediately prior to their procedure. These questionnaires were designed by the research team and include the three candidate primary outcome measures for a future trial alongside demographic questions recording their age, sex, ethnicity and partial postcode. The questionnaires can be viewed in Supplementary material 1. It will also be recorded whether the participant completed their planned treatment, where this information is available or provided by the participant.

In school settings, the young people will be given packs to take home which contain the screening cover letter, participant information sheets, consent and assent forms and baseline questionnaires. Those who wish to take part will be asked to complete and return all paperwork to the School Office or a similar secure location where it will be collected by the research team. Young people will be provided with a second questionnaire immediately prior to their vaccination *if* this can be done while maintaining confidentiality from peers. If this cannot be achieved, students will be emailed a copy to complete 1 day prior to their procedure.

Data will be collected about needle fear, completion of planned treatment and engagement with the resource. All participants will receive a £10 thank you voucher for their time. Recruitment will continue until the sample size is achieved.

### Inclusion and exclusion criteria

#### Inclusion criteria

- Children who require a needle-based procedure as part of their routine medical or dental care and who score as anxious of needles on screening
- Children aged 11- to 16 years

#### Exclusion criteria

- Children and parents/carers who lack capacity to give consent or assent
- Children with diagnosed concomitant anxiety disorders as identified by their medical history
- Children who are unable to communicate their thoughts or feelings for any reason (such as severe learning difficulties)
- Children and parents/carers who are involved in any other research which may impact this study

### Data analysis

All data will be entered into an electronic database (IBM® SPSS® Statistics v29). The quantitative outcomes for this feasibility study include recruitment rates, completion rates, and ability to acquire necessary data at baseline and follow-up visits. These will be analysed with simple descriptive statistics.

The feasibility study also aims to determine the most appropriate primary outcome measure for a future trial from three candidate measures—the VAS, CFS and IPS-Anx C. These will be analysed through simple descriptive statistics, validity testing (correlation coefficients) and reliability testing (Cronbach’s alpha) to evaluate their performance.

## Qualitative component

### Settings and sample size

Recruitment for this element of the study will comprise the same three sites. For this component of the study, qualitative interviews will be undertaken in order to explore the experiences of the young people who participated in the feasibility study and their parent/carer, as well as the experiences of healthcare professionals who assisted with recruitment or provided treatment to young people who participated.

### Young people and parent/carers

The interviews aim to explore their experience of participating in the study, their willingness to be randomised to a future study exploring the effectiveness of the resource and their thoughts on the acceptability of the resource.

### Healthcare professionals

The interviews aim to explore their experiences of either recruiting or treating young people in the study, and their willingness to recruit and randomise to a future trial.

It is likely that for the qualitative element of the study around 55 participants, comprising 20 young people, 20 parent/carers and 15 healthcare professionals will be sufficient. Similar studies to develop resources with dentally anxious children suggest that these sample sizes will be appropriate [25–27].

### Recruitment

Recruitment of young people and families will be undertaken from those who participated in the quantitative element of the study. Consent will be taken to contact the families to invite them to attend a subsequent qualitative interview. Care will be taken to aim to include a sample of participants from across the sites, including those who did and did not engage with the resource, and those who did and did not accept their procedure. Further participant information sheets will be provided with the invitation, and consent and assent taken if participants wish to undertake an interview.

Recruitment of healthcare participants will be undertaken to include a sample of those involved in recruitment to the study and those who treated study participants.

Semi-structured interviews with a topic guide will be undertaken to explore the participants’ experiences of involvement in the study, and to gain insight into acceptability of being recruited/recruiting and randomised/randomising in future, and of the resource itself. These will be recorded on a secure audio recorder and transcribed verbatim by an external company. All identifying information will be removed to ensure anonymity.

Interviews will be completed by one trained researcher. Participants will be able to select from telephone, virtual or face-to-face interview at a location of the participant’s choosing and will last between 30 and 45 min. It is not expected that these interviews will carry any material risks to participants; however, they may experience some distress in discussing their fear of needles. The interviewer has appropriate training and experience to support the young people should this occur, and breaks will be offered at any time [28]. Participants may also withdraw from the interview at any time.

All participants will receive a £10 thank you voucher for their time.

### Inclusion and exclusion criteria

#### Young people

All young people who participate will be eligible for inclusion with the exception of any who withdraw or decline to be contacted.

#### Healthcare professionals

All healthcare professionals involved in recruitment or treating participants will be eligible with the exception of any who are unable to communicate their thoughts and feelings for any reason.

### Data analysis

The semi-structured interviews will be recorded, transcribed verbatim and analysed using a framework analysis based on the Five Areas model of CBT [21]. To improve quality and trustworthiness of the analysis, two researchers will be involved at each stage of analysis.

### Success criteria

Success criteria are described in Table 1 below using green, amber and red criteria. Green indicates that progression to definitive trial is appropriate with no major amendments; amber that the trial may be feasible but with significant amendments and red indicates that a trial is unlikely to be feasible. For the study to be deemed successful, all outcomes must meet amber or green criteria.

**Table 1:** Success criteria for progression to definitive trial

Outcome	Green criteria	Amber criteria	Red criteria
Sites	All three sites recruited, and protocol delivered	At least two of three sites recruited, and protocol delivered	Less than two sites recruited, significant problems delivering the protocol
Participant recruitment	All three sites recruiting participants with no significant barriers encountered (at least 50% of target per site)	At least two sites able to recruit participants without significant barriers encountered (at least 50% of target per site)	Major barriers at more than one site
Willingness to be randomised to future trial	No significant barriers identified by young people in the qualitative interviews	Some barriers identified, but surmountable with changes to study design	Insurmountable challenges in randomising to future trial
Willingness to recruit to future trial	No significant barriers identified by healthcare professionals in the qualitative interviews	Some barriers identified, but surmountable with changes to study design	Insurmountable challenges in recruitment to future trial

### Outcomes without specific success criteria

These are uncertainties which are important to explore to inform the design of a future trial, but do not have set success criteria. This applies to the variability of the candidate primary outcome measures and the acceptability of the resource.

Variability of candidate primary outcome measures (VAS, CFS and IPS-Anx C) will be determined through analysis as described above, and calculation of standard deviations. This will help to inform the choice of future primary outcome.

Acceptability of the resource to participants and healthcare professionals will be determined through qualitative interview, and it should be determined if the resource is considered acceptable in its current format or if additional changes are required to improve it further.

## Timeline

A timeline for the study as per the SPIRIT 2013 checklist [29] can be seen in Table 2. This timeline may be subject to amendment depending on the vaccination schedule for the school setting or extended to allow for further recruitment of participants if required.

**Table 2:** Timeline of study

Timepoint	Enrolment quantitative element (December 2023–April 2024)	After consent	Prior to needle based procedure (max 1 day before)	After needle based procedure	Enrolment qualitative element (December 2023–June 2024)	After consent
**Enrolment:**						
**Eligibility screen**	X				X	
**Informed consent**	X				X	
**Baseline Questionnaire**		X				
**Invitation to interview**				X		
**Interventions:**						
**Access to resource**		X				
**Qualitative interview**						X
**Assessments:**						
**Demographics**		X				
**Children’s Fear Scale**		X	X			
**Visual Analog Scale**		X	X			
**Injection Phobia Scale-Anxiety**		X	X			
**Success of procedure**				X		

## Discussion

The self-help CBT resource has been developed to address a lack of self-help tools in the existing resources available to young people with fear of needles. Fear related to needles may cause negative health impacts for young people, and place additional stress on healthcare professionals involved in providing their care, and on the health service itself. The study presented in this protocol proposes to assess the feasibility of undertaking a future definitive RCT to evaluate the resource in the reduction of needle fear in young people aged 11–16 years. The study will provide methodological evidence addressing uncertainties around recruitment rates and numbers, retention rates, the ability to undertake such a protocol in school and secondary healthcare settings, the most appropriate outcome measures, the willingness of clinicians to recruit and participants to be recruited. It will also provide valuable insight into the acceptability of the resource to young people their parent/carers and healthcare professionals. It is expected that there will be some challenges to recruiting the school site, and that there will be evidence gained to support the use of one primary outcome measure. The other results are less easy to predict, but the authors expect to gain sufficient evidence for future trial. It is hoped that through the co-design collaboration, where young people have been engaged as partners in creating and designing the resource, that the resource will be more contextually appropriate.

The aims and methodology of this overarching study, including development of the resource, were designed with input from patients and the public which included healthcare professionals, young people aged 9–18 years old and parents/carers [30]. The Patient and Public Involvement [PPI] group were also integral in determining target age range, with 11–16 years being suggested as most appropriate.

Should the self-help resource prove acceptable, and the study procedures are found to be acceptable and feasible, the resource will be evaluated in a definitive trial, which will assess both clinical outcomes and cost-effectiveness. The feasibility study will contribute to the design of the future RCT by providing evidence which will help particularly in developing the recruitment plan for both sites and participants, and identification of the most appropriate primary outcome measure. The definitive trial will primarily assess the ability of the resource to improve coping skills and/or reduce needle fear in young people, and its cost-effectiveness. The likely design will be a mixed-methods parallel randomised trial undertaken in primary dental, medical and school settings. The complementary qualitative and quantitative elements will allow for the additional assessment of mechanisms of change and how the resource effects may vary with context. The eventual aim is to facilitate access to the resource nationally.

## Supplementary Information

Supplementary Material 1. CYP baseline questionnaire.

## Data Availability

The dataset which will be used and analysed during this study will be available in the University of Sheffield data repository. Data will be available following publication of study results.

## Abbreviations

CBT: Cognitive Behavioural Therapy
CFS: Children’s Fear Scale
IPS-Anx: The Injection Phobia Scale-Anxiety
NHS: National Health Service
NICE: National Institute for Health and Care Excellence
PPI: Patient and Public Involvement
RCT: Randomised controlled trial
VAS: Visual Analog Scale

## References

[1] McLenon J, Rogers M. The fear of needles: a systematic review and meta-analysis. J Adv Nurs. 2018;75(1):30–42. 10.1111/jan.13818. 30109720

[2] Kleinknecht RA, Lenz J. Blood/injury fear, fainting and avoidance of medically-related situations: a family correspondence study. Behav Res Ther. 1989;27(5):537–47. 10.1016/0005-7967(89)90088-0. 2818416

[3] Fu AZ, Qiu Y, Radican L. Impact of fear of insulin or fear of injection on treatment outcomes of patients with diabetes. Curr Med Res Opin. 2009;25(6):1413–20. 10.1185/03007990902905724. 19422281

[4] Sokolowski CJ, Giovannitti JA, Boynes SG. Needle phobia: etiology, adverse consequences, and patient management. Dent Clin North Am. 2010;54(4):731–44. 10.1016/j.cden.2010.06.012. 20831935

[5] Taddio A, Ipp M, Thivakaran S, Jamal A, Parikh C, Smart S, et al. Survey of the prevalence of immunization non-compliance due to needle fears in children and adults. Vaccine. 2012;30(32):4807–12. 10.1016/j.vaccine.2012.05.011. 22617633

[6] Berge KG, Agdal ML, Vika M, Skeie MS. High fear of intra-oral injections: prevalence and relationship to dental fear and dental avoidance among 10- to 16-yr-old children. Eur J Oral Sci. 2016;124(6):572–9. 10.1111/eos.12305. 27689943

[7] Kortesluoma R-L, Nikkonen M. ‘I had this horrible pain’: the sources and causes of pain experiences in 4- to 11-year-old hospitalized children. J Child Health Care. 2004;8(3):210–31. 10.1177/1367493504045822. 15358886

[8] Dower JS, Simon JF, Peltier B, Chambers D. Patients who make a dentist most anxious about giving injections. J Calif Dent Assoc. 1995;23(9):35–40. 9051996

[9] Moore R, Brødsgaard I. Dentists’ perceived stress and its relation to perceptions about anxious patients. Community Dent Oral Epidemiol. 2001;29(1):73–80. 11153566

[10] Ives M, Melrose S. Immunizing children who fear and resist needles: is it a problem for nurses? Nurs Forum. 2010;45(1):29–39. 10.1111/j.1744-6198.2009.00161.x. 20137022

[11] Paediatrics RC. Guidelines for the ethical conduct of medical research involving children. Arch Dis Child. 2000;82(2):177–82. 10.1136/adc.82.2.177. PMC1718211 10648379

[12] Chatio S, Baiden F, Achana FS, Oduro A, Akazili J. Knowledge and perceptions about clinical trials and the use of biomedical samples: findings from a qualitative study in rural northern Ghana. PLoS ONE. 2016. 10.1371/journal.pone.0152854. PMC4817990 27035916

[13] James AC, James G, Cowdrey FA, Soler A, Choke A. Cognitive behavioural therapy for anxiety disorders in children and adolescents. Cochrane Database of Systematic Reviews. 2015;2020(11). 10.1002/14651858.cd004690.pub4. PMC6491167 25692403

[14] Kennerley H, Kirk J, Westbrook D. An introduction to cognitive behaviour therapy: skills & applications. London: SAGE Publications; 2017.

[15] Öst LG, Hellström K, Kåver A. One versus five sessions of exposure in the treatment of injection phobia. Behav Ther. 1992;23(2):263–81. 10.1016/s0005-7894(05)80385-5.

[16] Wright B, Tindall L, Scott AJ, Lee E, Cooper C, Biggs K, et al. One session treatment (OST) is equivalent to multi‐session cognitive behavioral therapy (CBT) in children with specific phobias (aspect): results from a national non‐inferiority randomized controlled trial. J Child Psychol Psychiatry. 2022;64(1):39–49. 10.1111/jcpp.13665. PMC10087411 35915056

[17] Overview: Common mental health problems: Identification and pathways to care: Guidance. [cited 2023 Aug 16]. Available from: https://www.nice.org.uk/guidance/cg123.

[18] Hedman E, Andersson E, Ljótsson B, Axelsson E, Lekander M. Cost effectiveness of internet-based cognitive behaviour therapy and behavioural stress management for severe health anxiety. BMJ Open. 2016. 10.1136/bmjopen-2015-009327. PMC4854011 27113231

[19] Porritt J, Rodd H, Morgan A, Williams C, Gupta E, Kirby J, et al. Development and testing of a cognitive behavioral therapy resource for children’s dental anxiety. JDR Clin Transl Res. 2016;2(1):23–37. 10.1177/2380084416673798. PMC5576043 28879243

[20] Gomes HS, Viana KA, Batista AC, Costa LR, Hosey MT, Newton T. Cognitive behaviour therapy for anxious paediatric dental patients: a systematic review. Int J Paediatr Dent. 2018;28(5):422–31. 10.1111/ipd.12405. 29984460

[21] Williams C, Garland A. A cognitive–behavioural therapy assessment model for use in everyday clinical practice. Adv Psychiatr Treat. 2002;8(3):172–9. 10.1192/apt.8.3.172.

[22] Teare MD, Dimairo M, Shephard N, Hayman A, Whitehead A, Walters SJ. Sample size requirements to estimate key design parameters from external pilot randomised controlled trials: a simulation study. Trials. 2014. 10.1186/1745-6215-15-264. PMC4227298 24993581

[23] Billingham SA, Whitehead AL, Julious SA. An audit of sample sizes for pilot and feasibility trials being undertaken in the United Kingdom registered in the United Kingdom Clinical Research Network Database. BMC Med Res Methodol. 2013. 10.1186/1471-2288-13-104. PMC3765378 23961782

[24] Sim J, Lewis M. The size of a pilot study for a clinical trial should be calculated in relation to considerations of precision and efficiency. J Clin Epidemiol. 2012;65(3):301–8. 10.1016/j.jclinepi.2011.07.011. 22169081

[25] McMurtry CM, Noel M, Chambers CT, McGrath PJ. Children’s fear during procedural pain: preliminary investigation of the children’s fear scale. Health Psychol. 2011;30(6):780–8. 10.1037/a0024817. 21806301

[26] Hulin J, Baker SR, Marshman Z, Albadri S, Rodd HD. Development of a decision aid for children faced with the decision to undergo dental treatment with sedation or general anaesthesia. Int J Paediatr Dent. 2016;27(5):344–55. 10.1111/ipd.12267. 27684707

[27] Morgan AG, Rodd HD, Porritt JM, Baker SR, Creswell C, Newton T, et al. Children’s experiences of Dental Anxiety. Int J Pediatr Dent. 2016;27(2):87–97. 10.1111/ipd.12238. 27376925

[28] Sheffield Teaching Hospitals Level 3 Safeguarding Children and Young People PALMS (e-learning).

[29] Spirit checklist. [cited 2023 Aug 16]. Available from: https://www.spirit-statement.org/.

[30] NIHR Funding Awards: Award 301503 [cited 2023 November 15] Available from: https://fundingawards.nihr.ac.uk/award/NIHR301503.

