# Supplementary material for: Protocol for a feasibility study of a self-help resource based on the principles of cognitive behavioural therapy to reduce needle fear in young people aged 11- to 16 years
Source: Pilot Feasibility Stud. 2026 Aug 14;12:161. doi: 10.1186/s40814-026-01893-9 (PMC13753145; doi:10.1186/s40814-026-01893-9)
Supplement: Supplementary file 1 [file 40814_2026_1893_MOESM1_ESM.pdf]

The University Of Sheffield.

NHS Sheffield Teaching Hospitals NHS Foundation Trust

FUNDED BY NIHR | National Institute for Health Research

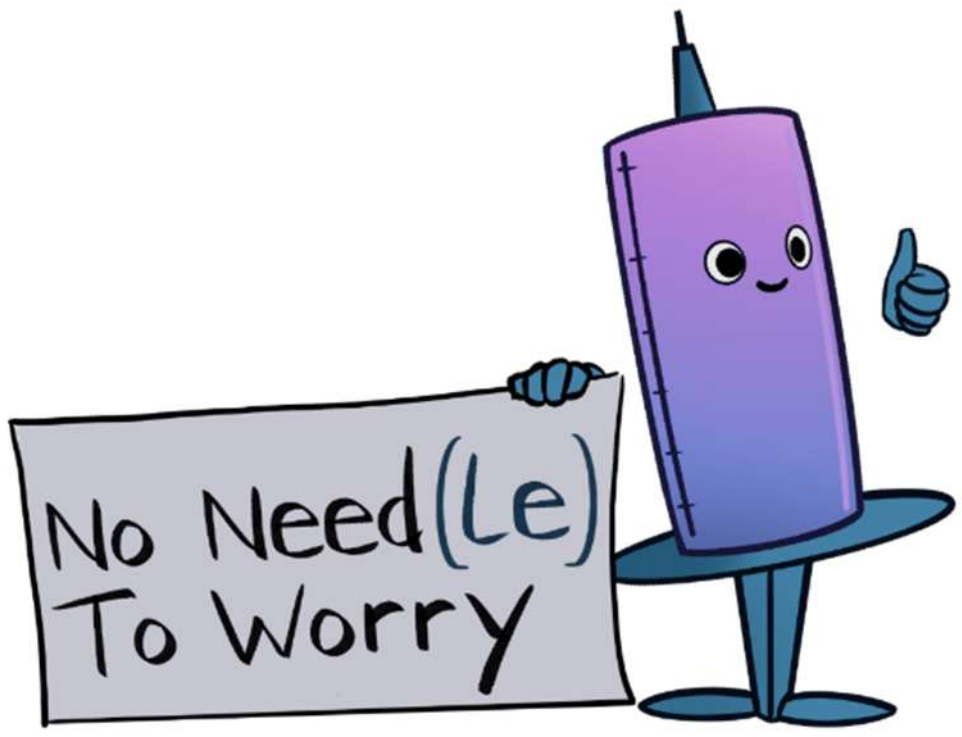

Research team to complete	
**Site ID:**	
**Participant ID:**	

No Need(le) To Worry: helping to make having needles easier for children and teenagers

Young Person First Questionnaire Healthcare

Instructions

Thank you for taking place in the project. We would like you to:

☺ Answer the questions

☺ Remember there are no right or wrong answers! We just want to know how you feel

☺ Give this booklet back to the person who gave it to you

The University Of Sheffield. NHS Sheffield Teaching Hospitals NHS Foundation Trust. FUNDED BY NIHR | National Institute for Health Research

The first questions are about how you feel about needles

1. Have you ever had treatment with a needle before?

Yes ☐

No ☐

If you answered 'Yes', please tick ☑ which of these you have had before:

Treatment	Had it before?
Vaccinations (e.g. an injection of medicine to stop you from getting sick/poorly)	☐
Getting an intravenous injection (e.g. a needle in your vein that connects to tubes and a bag of medicine)	☐
Having a blood test (e.g. a needle in your vein and blood withdrawn)	☐
Having a numbing injection at the dentist	☐
Giving a blood sample by having your finger pricked	☐

2. How do you feel when you need to have a needle? Please circle one of the faces below:

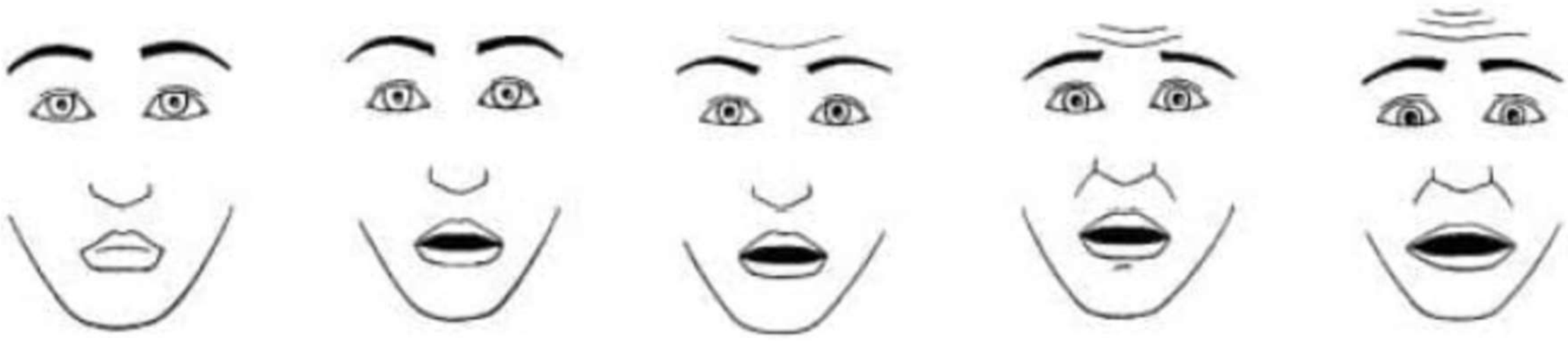

3. How do you feel when you need to have a needle? Place a mark along this line to show how you feel

Not at all worried — Extremely worried

4. **Below are a number of statements that young people use to describe their worry about having needles and injections.**

Read each carefully and tick the number that best describes your worry.

There are no right or wrong answers.

Please answer all items.

How worried would you feel about:

Statement	0 Not worried	1 A little bit worried	2 Somewhat worried	3 Very worried	4 Extremely worried
Giving a blood sample by having your finger pricked	☐	☐	☐	☐	☐
Having a needle in your arm	☐	☐	☐	☐	☐
Looking at a picture of a needle	☐	☐	☐	☐	☐
The smell of a hospital	☐	☐	☐	☐	☐
Having a numbing injection at the dentist	☐	☐	☐	☐	☐
Having a blood test (e.g. a needle in your vein and blood withdrawn)	☐	☐	☐	☐	☐
Watching another person have a blood test	☐	☐	☐	☐	☐
Getting an injection in your bottom	☐	☐	☐	☐	☐
Looking at a picture of a person having a needle	☐	☐	☐	☐	☐

How worried would you feel about:

Statement	0 Not worried	1 A little bit worried	2 Somewhat worried	3 Very worried	4 Extremely worried
Listening to someone talking about injections	☐	☐	☐	☐	☐
Looking at and touching the veins in your arm	☐	☐	☐	☐	☐
Watching a video about a person getting a needle	☐	☐	☐	☐	☐
Watching another person getting a needle	☐	☐	☐	☐	☐
Watching a person in a nurse uniform	☐	☐	☐	☐	☐
Having your ears pierced	☐	☐	☐	☐	☐
Getting a vaccination (e.g. an injection of medicine to stop you from getting sick/poorly)	☐	☐	☐	☐	☐
Getting an intravenous injection (e.g. a needle in your vein that connects to tubes and a bag of medicine)	☐	☐	☐	☐	☐
Watching another person having their finger pricked	☐	☐	☐	☐	☐

These questions are all about you, please complete or tick the boxes

5. What is your postcode?

6. Choose one section from A to F and tick the box that best describes your ethnic group

**A – White**	Welsh/ English/ Scottish/ Northern Irish/ British	☐
	Irish	☐
	Gypsy or Irish Traveller	☐
	Any other White background (please write in the box)	
**B – Mixed**	White and Black Caribbean	☐
	White and Black African	☐
	White and Asian	☐
	Any other Mixed background (please write in the box)	
**C – Asian**	Indian	☐
	Bangladeshi	☐
	Pakistani	☐
	Chinese	☐
	Any other Asian background (please write in the box)	
**D – Black**	Black Caribbean	☐
	Black African	☐
	Any other Black background (please write in the box)	
**E – Other**	Arab	☐
	Any other background (please write in the box)	
**F – Prefer not to say**	Prefer not to say	☐

7. Which of these best describes your gender?

Female/Girl ☐

Male/Boy ☐

Prefer not to say ☐

Other (please write) ……………………………………

Thank you very much for your time
